# Nurse-parent communication and interaction during caregiving events in single-family room neonatal intensive care units

**DOI:** 10.1016/j.pecinn.2026.100484

**Published:** 2026-07-02

**Authors:** Lene Tandle Lyngstad, Lena Gunterberg Heyn, Bente Silnes Tandberg, Hanne Aagaard

**Affiliations:** a MNSc, Department of Pediatrics and Adolescent Medicine, Drammen Hospital, Vestre Viken Hospital Trust, Drammen, Norway Department of Master and Postgraduate Education, Lovisenberg Diaconal University College, Oslo, Norway Department of Nursing and Health Sciences, University of South-Eastern Norway, Drammen, Norway; b Prof University of South-Eastern Norway, Centre for Health Technology, Department of Nursing and Health Sciences, Drammen, Norway; c PhD, Department of Pediatrics and Adolescent Medicine, Drammen Hospital, Vestre Viken Hospital Trust, Drammen, Norway Department of Master and Postgraduate Education, Lovisenberg Diaconal University College, Oslo, Norway; d Prof Department of Master and Postgraduate Education, Lovisenberg Diaconal University College, Oslo, Norway

**Keywords:** Communication, Interaction, Single-family room, Parents, Nurse, Family-centred care, Family-centered care, Neonatal intensive care units

## Abstract

**Objectives:**

This study systematically describes neonatal nurses' communication and interactions with parents of preterm infants during nurse-guided caregiving events in the first week after birth, in family-centered neonatal intensive care units with single-family rooms.

**Methods:**

In-situ video observations were analysed using an interaction analysis approach.

**Results:**

Six nurses and eleven parents of seven preterm infants participated in the study. Six video observations were conducted in two NICUs, each lasting 25–65 min. The analysis suggested five communication patterns, coded as *progress, sensitive & empathic communication, supervision & teaching, positive feedback,* and *correction,* and two themes—*dual roles* and *micro-interactions*—based on the nurse's communication and nurse–parent interactions.

**Innovation:**

This study focused on interactions in family-centered neonatal intensive care units with single-family rooms. The in situ video observation of nurse-guided caregiving events provides a new and important perspective on the implementation and operation of single-family room design in neonatal intensive care units.

**Conclusion:**

Our study suggests that nurse–parent communication and interactions during caregiving events are complex and multifaceted. Nurses must balance the provision of advanced care to preterm infants with support for parents. The implementation of family-centered care and single-family room design has positive outcomes for both infants and parents, but it requires advanced communication skills in comprehensive interactions with parents. We recommend that the implementation of family-centered models of care and single-family rooms in new NICUs in the future be accompanied by a broader focus on communication with parents as a core component of care.

## Highlights

• Early parental involvement in caregiving activities is a balancing act for the nurses working in family-centred SFR NICUs.
• The early caregiving event is a complex intervention where the parents hold dual roles as caregivers and care recipients.
• Communication training should be viewed as an integral part of the education and professional training provided to neonatal nurses.
• The study method with in-situ video observationis innovative and provide new knowledge of nurse-parents communication.

## 1 Introduction

Communication is an important tool and key component in nurse-parent interactions within single-family room (SFR) neonatal intensive care units (NICUs) [1]. Providing care for the preterm infants and their parents requires enhanced medical competence and relational skills as the nurses are both clinical caregivers for the infants and relational anchors for the parents [2]. How nurses communicate with the parents affects parent-related outcomes, including parental coping and participation in care [3]. Adapted communication is important for achieving optimal parent-staff relationships and parent self-management [2], [3], [4].

The implementation of SFR design in Norway [5] and worldwide [6], is driven by the importance of a low-noise and low-light sensory environment to stimulate the preterm infants' brain growth and address their early developmental needs [7]. SFR NICUs encourage 24/7 parental presence through accommodations such as private beds and toilet facilities [8], [9], which have been shown to reduce parental stress [10], increase parent-infant closeness [11] and improve parents` coping [12]. A family-centered model of neonatal care (FCC) has been adopted in many countries over the past decade, including Norway [13], [14]. As a theoretical framework, FCC seeks to position parents as integral partners in the care of their infants, emphasising respect, information sharing, participation and collaboration as its principles [15]. FCC is associated with reduced mortality [16], improved motor, cognitive and behavioural development [17], shorter hospital stays and higher parental satisfaction [18]. The model also increases the provision of emotional support to parents as part of the care given to the family [19], including interventions to improve parent-staff communication [1], thus in practice, the degree of parental support varies [20], [21]. Davidson et al. presented guidelines for FCC [20] that included family presence, family support and communication with family members, highlighting the need for training in communication techniques for the NICU staff. The implementation of SFR and FCC therefore entails new and advanced communication competence demands for nurses [1], [8].

Previous research has focused mostly on communication barriers and facilitators' [22], [23] and the parents´ and nurses´ experiences [24], [25], leaving a description of the actual communication between the nurses and parents unexplored [25], [26], [27]. A more comprehensive understanding and thorough insight may contribute to improvements in nurse-parent communication and interaction in the future.

This study aims to explore and describe nurse-led communication and interaction with parents during caregiving events. The research question that guided the study were as follows: What characterises the nurses´ communication and interaction during guided caregiving events with parents in family-centered SFR neonatal units?

## 2 Design and methods

### 2.1 Study design

This observational study adopted a qualitative exploratory descriptive design with video recordings. It is based on the assumption that both parents are invited to act as caregivers during hospitalisation in a family-centered NICU [21]. Video observation was selected as the research method because it allows for repeated viewings of naturally occurring nurse-parents communication and interaction, and it enables evaluation of what the parties say rather than what they remembered or imagined saying [28], which are impossible with other methods [29].

### 2.2 Setting and participants

The study was conducted in two Norwegian NICUs with a family-centered care model in single-family rooms. Each unit had approximately 5000 births per year. Unit 1 was a 17-bed, level III NICU that provided intensive care for preterm infants born from 28 weeks' gestation and infants with medical conditions that require specialised medical care. The other unit was a 18-bed level IV NICU with preterm infants born from 23 weeks ‘gestation and those that required surgery and/or advanced life support.

The nurses and Norwegian speaking parents of all hospitalised preterm infants born between 23 and 30 gestational weeks in the two family-centered SFR units were invited to participate in the study with a convenience sample approach [30]. The first author presented the study to the nursing staff in both units before the project began. As new families became eligible for participation, the parents were recruited and included with successive enrolment in collaboration with the head nurses. When the parents confirmed their interest, the first author approached them and made an appointment to sign the written consent form. The nurses were recruited continuously. If a nurse declined to participate, the first author approached a new nurse on the next shift. Eight families were invited to participate and six volunteered. One family in each unit declined to participate. In one unit, all the invited nurses volunteered to participate, while in the other, five nurses declined due to either work overload or because they deemed the video recording method to be intrusive.

Six nurses and 11 parents of seven very or extremely preterm infants were successively recruited to participate in the study. One family was represented by the father. Six video observations were conducted from August to December 2022 (Unit 1) and April to August 2024 (Unit 2). The observations lasted from 25 to 65 min (mean duration:43 min). Demographic data were collected about the infants, from the parents and nurses (Table 1).

**Table 1:** Characteristics of the infants, parents, and nurses.

Variables	Unit 1	Unit 2
**Infants**	n = 3	*n* = 4⁎
Gestational age/weeks	28 (28–30)	26 (25–29)
Median (min–max)		
Birth weight/g	1310 (1180–1460)	857 (830–1635)
**Infants on breathing support**		
Mechanical ventilator		n = 1
DuoPAP	n = 1	*n* = 1
CPAP	*n* = 2	*n* = 2
Length of stay/weeks	12 (6–16)	10 (8–12)
**Parents**	*n* = 5	*n* = 6
Mothers age/years	37 (32–42)	31 (29–32)
Fathers age/years	29 (29–52)	32 (32)
**Educational level**		
High school	1	4
College/university	4	2
**Nurses**	n = 3	*n* = 3
Age/years	48 (36–51)	32 (32–52)
Higher educational level/years	5	5
NICU experience/years	12 (10–19)	8 (1–28)

⁎ One family had twins.

### 2.3 Data collection

Two surveillance cameras were installed in the room. The first camera recorded an overview of the room while the second camera focused on the infants´ incubator and captured the nurses´ and parents´ nonverbal facial expressions, posture and body language. The nurse and one of the parents wore a wireless microphone. The technical equipment was especially suitable for the NICU environment with minimal lighting and noise to avoid any negative impact on the preterm infants´ well-being. The caregiving event was chosen as the scenario for the recordings because it is a common nurse-guided activity in the NICU.

Both of the participating units performed developmental care for each individual infant [31], hence the video recordings showed events based on the infants` needs and not standardised care procedures [32], [33]. A caregiving event consists of several steps (Table 2), including diaper change and weighing, which are stressful procedures for preterm infants [34], [35]. The nurse considers whether a full procedure can be conducted based on the infants´ signals before and during the event, which explains why the video recordings vary in their length and content.

**Table 2:** Example of a caregiving event framework.

1.	Approach and wake up the infant
2.	Wash the face and body
3.	Skin observation and care
4.	Diaper change
5.	Weighing
6.	Position or place the infant skin to skin with the parents

### 2.4 Data analysis

The analysis was inductive, data driven and inspired by Jordan and Henderson's interaction analysis (IA) [36]. The analysis is grounded in the premise that video recordings function as empirical traces of interaction that permit analytic validation. IA aims to identify regularities and patterns in the videorecorded interactions, and to understand how the participants make sense of each other's actions as meaningful, orderly and projectable. In addition, the analysis applies a triadic analytic lens, because the interaction unfolds between three parties, the nurse, the parents and the infant as the shared focus of attention [37].

First, the videos were watched several times and transcribed descriptively for both verbal and nonverbal communication by the first author, using an Excel spreadsheet. Then a content log (Table 3) was created to obtain an overview of what was happening and to search for patterns in the observed behaviours [36]. Codes were created during this process as shown in Table 4. Finally, two themes were conceptualised after a critical review of the video recordings and analysis.

**Table 3:** Example of a content log.

Time	Nurse (communication)	Parents (communication)	What is happening (content)	Preliminary codes
00:00:10	We are now going to wash him, change the diaper and in the end, we weigh him. I think that one of you can wash him while the other supports him.	Father: Yes	The nurse approaches the parents in the SFR and explains what they are going to do during the daily care event. The nurse is gesturing with her hands and smiling at the parents.	Progress (Initiating and ending events)
	He feels safe if you hold him steady with your hands.		The nurse is gesturing with her hands and smiling.	
	And then we will weigh him in the end.	Mother: Yes		Supervision and teaching
	Shall we start with the head and work our way down?	Mother: Yes		Progress
00:01:39	First, you can pour warm water in a bowl and bring it to the incubator.	Father: Yes	The nurse is pointing at the bowl on the shelf over the sink.	Progress

**Table 4:** Codes describing patterns of the nurses' communication.

Code names	Progress	Sensitive & empathic communication	Supervision & teaching	Positive feedback	Correction
Description of the nurse's verbal and nonverbal communication	How the nurse initiates and closes an event	Positive reactions Emotional support Attentive communication Positive nonverbal utterances: laughs smiles and nodding	Explanations Practical supervision and information (i.e., clarification and/or confirmation)	Direct feedback in the form of positive evaluation, compliments, or praise	Negative feedback Derogatory behaviour Nonverbal utterances Ignored communication
Total of observed episodes	95	54	138	64	8
Examples	Nurse: Now I think we finish up and position him to sleep. How do you think he would like to be positioned? Or do you want to care for him skin-to-skin maybe? What is your plan? Father: We will wait and do it later. Nurse [to the father]: Okay. Maybe put him in a prone position? Father: Yes. Nurse: Do you want to try? Father: No, you can do it.	Mother: My feet hurt badly so I don't want to take her out from the Incubator myself. Nurse: Oh, I understand. Of course. I understand that very well. I want you to lift and hold her to your chest, because then she feels safe, but it's totally up to you, we can wait until you feel ready.	Nurse: Do you see how he is starting to move? It's nice to give him some time to wake up, right? And not just start to wash him immediately after he wakes up from deep sleep. Mother: How do you know when he is in deep sleep? Nurse: He is in deep sleep when there are no movements at all.	Nurse: Wonderful! As I would have done it myself. It looks perfect! Really good! It is nice to see how he [the infant] is enjoying what you do. It means that you two are doing a good job!	Nurse [to the mother]: Did you wash properly on the side? Or did you only wash him on the back? […] You have to wash him thoroughly. See that you unfold the cloth and use the whole surface and wash with long and slow movements, not the small ones that you do.

The co-authors watched the videos independently and discussed the content continuously during the data analysis. Divergent interpretations were openly examined until a shared understanding was reached. Patterns of communication and central categories of interaction were extracted, discussed and reflected on by the research group, to ensure transparency, credibility and trustworthiness. The use of video recordings contributed to the study's dependability by allowing the group to review the data throughout the analysis and ensure that the interpretations were based on the original content [38].

The evidential basis of the data was strong as the video recordings provided rich material with numerous communicative events [39]. Limiting the dataset to six video recordings ensured that each interaction can be examined in detail to capture its complexity, while maintaining analytic feasibility. Furthermore, the interaction analysis involves analysing each recording as a dense, multilayered sense of meaning and having fewer recordings allowed for a deeper and more rigorous analysis [39].

### 2.5 Ethical considerations

The first author sat in the hallway outside the SFR, and watched the events on a computer, both to avoid interference with the participants during the recording and to ensure that the observation could be paused or stopped in the case of acute life-threatening episodes or other unforeseen events. The study participants were considered vulnerable at the time of the video recordings, which was an important ethical issue and necessitated careful consideration and adjustments throughout the project [40]. The first author ensured that the video recordings were adjusted as much as possible to meet the participants´ needs. The infants were either very or extremely preterm, and the parents displayed their early care interventions with their infants on camera. The nurses, who were the main objects of observation, were exposed because communication is both deeply personal and a professional competence [41]. Hence the first author engaged in close dialogue with the parents and the nurses before and after the video recordings to ensure their comfort [40].

The project was approved by the national data protection for research services (SIKT) (Reference no: 638661) and the personal protection representatives (PVO) (Reference no: 22/08882–2/294219).The data will not be made available due to concerns about participants confidentiality and in accordance with the Norwegian Health Research Act [42].

## 3 Results

The analysis resulted in five codes (Table 4), as described in Section 3.1 and two themes as explained in Section 3.2.

### 3.1 Nurse communication

We identified five codes to describe how the nurses communicated with the parents: *Progress, Sensitive & Empathic Communication, Supervision & Teaching, Positive Feedback and Correction* (Table 4). The coding contributed to identify patterns in the nurse's communication. The content of the caregiving events followed the same order in all observations. The nurses approached the parents, explained what was going to happen and described their expectations. All the nurses took the lead initiating and ending each event. They gave the parents the opportunity to play an active part in the caregiving by letting them make choices for themselves and the infant, and they supervised the parents in practical handling and care.

The nurses provided positive feedback directly tied to the parents' performance. Most of the nurses communicated in a consistently sensitive and empathic manner with only a few instances of corrective communication noted. The nurses` gaze, facial expressions and positive utterances, such as *“You did well” and “Very good”*, seemed to be important parts of the nurse's supportive and affirmative communication. They communicated indirectly to the parents through utterances to the infant such as”. It is so comforting to be touched by Mom and Dad” and “You like it when Dad holds his hands on your body”. In this way the nurses both communicated their interpretation of the infants` behaviour and supported the parents as they acted as caregivers.

### 3.2 Nurse-parent´ interactions

The parents participated in caregiving events for the first time, with the exception of simple nurse-guided diaper changes. Caregiving events are complex interactions with multiple participants, who communicate verbally and nonverbally with one another, often at the same time. The analysis indicated that the nurses simultaneously supported the parents in active caretaking, offered emotional support to the parents, and observed and supported the infant. The analysis of the interactions resulted in two themes: *dual roles* and *micro-interactions.*

#### 3.2.1 Dual roles

The recorded observations suggest that the nurses were in charge of and active participants in the caregiving event, while the parents shifted between being active (caregivers for the infant) and passive (care recipients of the nurses) participants. The nurses monitored and performed care for the infants while simultaneously supervising the parents. In addition, the nurses paid attention to the parent's personal needs and offered them practical and emotional support.

Nurse: *Can you remove the CPAP helmet and wash behind his ears?* (Nurse acting as a supervisor).

Father: *Yes. His head is so small and fragile, I am afraid that I will hurt him* (Father acting as a caregiver).

Nurse: *You can manage this, it's okay* (Nurse acting as a supervisor).

Father: *I hold his head like this and remove the helmet slowly* (Father acting as a caregiver).

Nurse: *Very good!* (Nurse acting as a supervisor).

Father: *Shall I continue to hold my hand under his head?* (Father acting as a caregiver).

Nurse: *No, it isn't necessary* (Nurse acting as a supervisor).

(The father stretches his back and holds his hands to his lower back).

Father*: Oh, it was good to stretch my back!* (Father acting as a care recipient).

Nurse: *I didn't know that it was so uncomfortable for you. I am sorry! Do you want me to raise the incubator for you?* (Nurse acting as a caregiver).

The next example demonstrates how nurses act as active participants who focus on the infant, practical handling and supervision, and the mothers´ personal needs.

Nurse: *Can you raise the (incubator) roof? It's a button here. And then, it's nice if you can hold your hands on her while you do it.* (Nurse acting as a supervisor).

Mother: *Yes* (She holds her hand on the infant and pushes the button). (Mother acting as a caregiver).

(The nurse sees the mother holding her hands on her back).

Nurse: *Do you want to sit down on a stool?* (Nurse acting as a caregiver).

Mother: *Yes, my back hurts if I stand up too long, after my C-section* (Mother acting as a care recipient).

Nurse: *I can see that. Of course, no problem. Do you want me to lower the incubator for you?* (Nurse acting as a caregiver).

#### 3.2.2 Micro-interactions

Further scrutiny of the nurse-parent communication indicated different layers of micro-interactions happening simultaneously between the parties, with the participants communicating with the other entities simultaneously. In the next example, the parents and the nurse stand around the infant in the incubator looking at him, and then they gently wake him up. The parents touch the infant and communicate with the nurse, each other and the infant, meanwhile the nurse observes and supervises the parents in parallel.

Mother (to the infant): *You are like your dad, refusing to wake up.*

(The nurse giggles in response to the mother-infant communication).

Father (to the infant): *Did you sleep well?*

Nurse (on behalf of the infant): *Yes.*

Mother (to the infant): *Little sweetie, are you going to wake up?*

Father (on behalf of the infant): *No, I don't want to wake up.*

Nurse: *There's some movement here. Do you see how he is starting to move? It's nice to give him some time to wake up before we move on.*

Mother: *How do we see when he is in deep sleep* versus *light sleep?*

Nurse: *When he is in deep sleep his face does not move and when he moves towards light sleep, you can often see movements behind the eyelids.*

Nurse: *Let us start to gently remove the CPAP head gear and wash the face.*

In the next example, the nurse observes the mother-infant interaction closely, while simultaneously monitoring the technical equipment as the mother talks to the infant and turns her around. The nurse smiles and giggle in response to the mother's interaction with the infant. The nurse supervises and responds concurrently with direct positive and sensitive feedback on the mothers` handling of the infant.

Mother (before the diaper change): *Do you want me to turn her around or is it better this way?*

Nurse: *You can decide but I think that changing the diaper in a sideways position is best for her because then she feels safe and supported*.

Mother (to the infant): *Do you like your pacifier? I think you are becoming a pacifier-girl just like your older sister.*

Nurse: *It is very nice for her when you support her with both your hands like this.*

## 4 Discussion

This study explored how nurses communicate and interact with parents of preterm infants during the first nurse-guided caregiving events in family-centered SFR NICUs. Two overarching findings emerged. First, parents occupy dual roles during caregiving events, functioning both as caregivers for their infant and as care recipients with personal emotional and informational needs. Second, nurses' communication followed identifiable patterns that displays caregiving events as complex, relational interventions composed of multiple simultaneous micro-interactions. Previous research has highlighted that parental involvement in infant care is often accompanied by emotional vulnerability and a substantial need for acknowledgement and emotional support, particularly during caregiving activities in the early, critical NICU phase [24], [25]. However, the coexistence of caregiving responsibility and dependency within the same situated interaction has not been explicitly conceptualised. The recognition of parents' dual roles shows how the nurse-parent relationship extends beyond task-oriented instruction to include continuous emotional attunement, relational support, and role negotiation. This duality introduces an inherent tension between parental agency and dependence [43]. Parents are encouraged to act as primary caregivers, yet simultaneously rely on nurses for reassurance, validation, and guidance. Such role ambiguity may be particularly salient during the first weeks, when parents are inexperienced, anxious, and adapting to the highly technological NICU environment [44]. Fegran et al.'s description of parents actively seeking closeness to the nurses during the acute stage further underscores the relational significance of these encounters [45]. The caregiving event thus becomes a pivotal arena where parental agency is fostered while vulnerability is managed, aligning with previous work highlighting parents' need for acknowledgement and emotional support [24], [25].

The five identified codes revealed consistent and recurrent patterns of communication (Table 4). Initiation and closure were characterised by structured communication, with nurses signalling responsibility by informing parents about the upcoming procedures and closing events with positioning instructions.

Supervision and teaching dominated the nurses' communication, reflecting nurse-guided caregiving within a family-centered care framework [20]. Through explanations and demonstrations, nurses supported parental involvement and the gradual transfer of caregiving responsibility. In line with previous research nurse-guided parental participation appears to strengthen parental sensitivity by supporting parents in interpreting and responding to their infant's cues, which is essential for both attachment, formation and neurodevelopmental outcomes [7], [46]. Structured supervision and teaching enhance confidence, reduces parental uncertainty, and strengthens self-efficacy, aligning with evidence linking communication quality to satisfaction and discharge preparedness [47], [48]. These findings highlight the nurses` expanded role as educators, requiring strong communication skills, and emphasise the need for organisational support to sustain effective FCC practices.

Empathic and sensitive communication permeated the supervision, feedback, and even task-oriented instruction. The nurses consistently responded to parents' actions and bonding attempts with the infant, with affirmative verbal and non-verbal cues, such as smiles, nods, and gentle laughter. Framing empathy as a quality embedded within multiple communicative practices reduces analytical redundancy while emphasising its integrative role in relational care. The nurses in this study possessed high levels of formal competence and extensive NICU experience, which may point to their consistent use of sensitive and empathic communication. Empathy and sensitivity appear to be cultivated through a combination of professional development, clinical experience, and tacit workplace learning, rather than solely through formal communication training [49]. Nevertheless, existing research shows that empathic practice may be constrained by workload, staffing models, and organisational priorities [43], [44], and that insufficient empathic responses can increase parental stress and hinder bonding [50]. The few exceptional cases, in which empathic responses were less evident, may point to the boundaries of relational practice with heightened clinical demands or contextual constraints, suggesting that empathic communication is sensitive to workload and situational pressure [51]. Examining such exceptions may indicate that empathy is not merely an individual attribute, but rather a practice shaped by organisational conditions and care culture [51], [52], [53].

Positive feedback emerged as a dominant and deliberate relational strategy. By affirming parents ‘caregiving actions, the nurses appeared to foster confidence, trust and emotional reassurance. Such trust-building communication aligns with core principles of family-centered care [20] and could be especially critical in SFRs, where parents are continuously present [54]. The significant use of affirmation may be interpreted as a means of regulating parental anxiety while maintaining asymmetrical responsibility for the medically fragile infants. The limited use of correction could point to an implicit power dynamic in which nurses` professional authority is exercised gently to support parental engagement rather than compliance. This balancing act illustrates the complexity of early parental involvement, where nurses hold medical accountability while encouraging parental participation [50], [55], [56].

Our findings also draw attention to nurses' invisible workload. Beyond observable technical tasks, nurses engage in continuous emotional regulation, anticipatory reassurance, and relational monitoring throughout the first caregiving events. This unrecognised work constitutes a significant component of nursing practice in family-centered NICUs, particularly where parents are continuously present and expectations of availability and partnership in care are high.

### 4.1 Strengths and limitations

This study has several limitations that must be considered when interpreting its findings. First, the study was conducted within a Norwegian institutional and cultural context. Norwegian neonatal care is shaped by broader Nordic care traditions including egalitarian norms, relatively flat authority structures and expectations of collaborative decision-making. These cultural features, together with national regulations, nurse-patient ratio and organisational arrangements in publicly funded NICUs, may have influenced the observed interactional patterns. As such, the findings described in this study may reflect characteristics specific to the Norwegian and Nordic contexts rather than to NICU practice in general, which limits the transferability of the findings.

Second, although the video-based methodology provides rich multimodal data, the analysis primarily focused on verbal and interactional structures. Nonverbal, embodied and emotional cues were only partially incorporated into the analysis. This selective focus may have constrained the interpretive depth, particularly regarding affective dynamics and subtle forms of alignment, discomfort and negotiation. Moreover, the predominance of positive and supervisory communication in the video data, with relatively few instances of overt tension or resistance, may raise questions regarding the presence of cameras influenced the interaction. However, previous research has found that nurses´ communicative behaviour is only influenced by video recording to a limited extent [40]. In addition, all the parents were proficient in the Norwegian language, which may be seen as a limitation in transferability to non-native speaking parents. Nurses' communication with parents who speak other native languages has its own complexities and needs to be explored in future research.

Finally, the absence of the parental perspective in this study represents an analytic limitation, nevertheless, the analytic scope was the nurse-led communication with parents and resulted in a detailed description of how the nurses communicated with the parents in real time during guided caregiving events. Future research incorporating parental voices and more systematic attention to embodied and emotional dimensions would strengthen the evidence and provide a more comprehensive understanding of these dynamics. Still, the findings provide a unique insight into the nurses` interactions with parents, that focuses on the actual, detailed communication and may be transferable to other nurse-patient contexts.

### 4.2 Innovation

This study contributes novel insights into family-centered neonatal care by advancing both methodological and conceptual understandings of nurse–parent communication and interaction in family-centered SFR NICUs. The methodological innovation lies in the use of close-up video observations to capture nurse-guided caregiving events as they unfold in real time. While much neonatal research relies on interviews or retrospective accounts, this real-time observational approach allows detailed analysis of verbal and non-verbal communication, moment-to-moment encounters, and relational micro-interactions that are otherwise difficult to access. Applying video-based methods to early caregiving events therefore represents an underutilised but highly informative approach in NICU research.

Conceptually, the study challenges prevailing, linear models of nurse–parent interaction by demonstrating that parents occupy dual roles as caregivers for their infant and as care recipients with emotional and informational needs. By empirically illustrating how these roles coexist within the same interaction, the study provides a more nuanced understanding of family-centered care as a dynamic and relational practice rather than a unidirectional process of parental involvement.

Furthermore, the study offers a novel perspective by conceptualising the first nurse-guided caregiving event as a complex relational intervention composed of multiple concurrent micro-interactions. These include supervision, instruction, emotional attunement, affirmation, and subtle regulation of parental anxiety. This complexity has received limited attention in previous research, which has tended to focus either on parental experiences or nurses' instructional roles.

An additional innovative contribution concerns the identification of nurses' communicative and emotional work as an invisible but substantial component of caregiving in SFR units. Parents of preterm infants require intensive practical and emotional support, particularly in the early phase after admission. The study points to how nurses continuously engage in relational and communicative labour alongside technical supervision, highlighting demands that are often overlooked in workload planning and organisational models of family-centered care.

Together, these findings offer a new analytical perspective in understanding nurse–parent communication in SFR NICUs and could have implications for professional education, training in relational communication, and the organisation of workload in family-centered neonatal intensive care units. By displaying the relational complexity of early caregiving events, this study contributes innovative knowledge with potential to enhance both quality of care and sustainable parental involvement.

## 5 Conclusion

The first caregiving events constitutes a central arena for nurse–parent partnering, where the nurse must navigate parents' dual roles while simultaneously supporting parent–infant bonding, handling and positioning. This aligns with research describing caregiving as consisting of multiple moment-to-moment verbal and nonverbal exchanges occurring in parallel between participants. The multifaceted demands placed on nurses during caregiving events therefore require not only clinical expertise, but also strong interpersonal skills. The development of nurses` professional identity, competence and interpersonal skills is essential for building an occupational foundation which allows nurses to connect, communicate and collaborate with parents. Our findings indicate that nurse-guided caregiving events are not merely instructional encounters but relational processes through which partnerships are negotiated and sustained. Parents' willingness to participate in early caregiving requires nurse initiative, sensitivity and sustained support. Recognising parents as both caregivers and care recipients emphasise the communicative demands placed on nurses in family-centered NICU care.

## CRediT authorship contribution statement

**Lene Tandle Lyngstad:** Writing – review & editing, Writing – original draft, Project administration, Methodology, Investigation, Formal analysis, Data curation, Conceptualization. **Lena Gunterberg Heyn:** Writing – review & editing, Supervision, Methodology. **Bente Silnes Tandberg:** Writing – review & editing, Supervision, Conceptualization. **Hanne Aagaard:** Writing – review & editing, Supervision, Conceptualization.

## Declaration of competing interest

The authors declare that they have no known competing financial interests or personal relationships that could have appeared to influence the work reported in this paper.

## References

[1] Franck L.S., O’Brien K. (2019). The evolution of family-centered care: from supporting parent-delivered interventions to a model of family integrated care. Birth Defects Res.

[2] Bry A., Wigert H., Bry K. (2023). Need and benefit of communication training for NICU nurses. PEC Innov.

[3] Labrie N.H., van Veenendaal N.R., Ludolph R.A., Ket J.C., van der Schoor S.R., van Kempen A.A. (2021). Effects of parent-provider communication during infant hospitalization in the NICU on parents: a systematic review with meta-synthesis and narrative synthesis. Patient Educ Couns.

[4] Willem-jan W.W., Lorie E.S., van Veenendaal N.R., van Kempen A.A., Ket J.C., Labrie N.H. (2021). The functions of adequate communication in the neonatal care unit: a systematic review and meta-synthesis of qualitative research. Patient Educ Couns.

[5] Grundt H., Tandberg B.S., Flacking R., Drageset J., Moen A. (2020). Associations between single-family room care and breastfeeding rates in preterm infants. J Hum Lact.

[6] van Veenendaal N.R., Heideman W.H., Limpens J., van der Lee J.H., van Goudoever J.B., van Kempen A.A. (2019). Hospitalising preterm infants in single family rooms versus open bay units: a systematic review and meta-analysis. Lancet Child Adolescent Health.

[7] Bergman N.J. (2019). Birth practices: maternal-neonate separation as a source of toxic stress. Birth Defects Res.

[8] Franck L.S., Cormier D.M., Hutchison J., Moore D., Bisgaard R., Gay C. (2021). A multisite survey of NICU healthcare professionals’ perceptions about family-centered care. Adv Neonatal Care.

[9] Kainiemi E., Hongisto P., Lehtonen L., Pape B., Axelin A. (2021). Effects of single family room architecture on parent–infant closeness and family centered care in neonatal environments—a single-center pre–post study. J Perinatol.

[10] Loutfy A., Zoromba M.A., Mohamed M.A., El-Gazar H.E., Andargeery S.Y., El-Monshed A.H. (2024). Family-centred care as a mediator in the relationship between parental nurse support and parental stress in neonatal intensive care units. BMC Nurs.

[11] Wielenga J., Pascual A., Ruhe K., Aarnoudse C., van Kaam A. (2025). Effect of shifting from open bay to single-family rooms on closeness in a NICU. Acta Paediatr.

[12] Tandberg B.S., Frøslie K.F., Flacking R., Grundt H., Lehtonen L., Moen A. (2018). Parent-infant closeness, parents’ participation, and nursing support in single-family room and open bay NICUs. J Perinat Neonatal Nurs.

[13] Lyngstad L.T., Le Marechal F., Ekeberg B.L., Hochnowski K., Hval M., Tandberg B.S. (2022). Ten years of neonatal intensive care adaption to the infants’ needs: implementation of a family-centered care model with single-family rooms in Norway. Int J Environ Res Public Health.

[14] Hodgson C.R., Mehra R., Franck L.S. (2024). Child and family outcomes and experiences related to family-centered care interventions for hospitalized pediatric patients: a systematic review. Children.

[15] Zgambo M., Blamires J., Foster M., Al-Motlaq M., O’Sullivan T.A., Houghton D. (2025). Child and family centred care: a three-phased principle-based concept analysis. J Adv Nurs.

[16] Örtenstrand A., Westrup B., Broström E.B., Sarman I., Åkerström S., Brune T. (2010). The Stockholm neonatal family centered care study: effects on length of stay and infant morbidity. Pediatrics.

[17] Raghupathy M.K., Parsekar S.S., Nayak S.R., Karun K.M., Khurana S., Spittle A.J. (2025). Effect of family-centered care interventions on motor and neurobehavior development of very preterm infants: a systematic review and meta-analysis. Phys Occup Ther Pediatr.

[18] Segers E., Ockhuijsen H., Baarendse P., van Eerden I., van den Hoogen A. (2019). The impact of family centred care interventions in a neonatal or paediatric intensive care unit on parents’ satisfaction and length of stay: a systematic review. Intensive Crit Care Nurs.

[19] Roué J.-M., Kuhn P., Maestro M.L., Maastrup R.A., Mitanchez D., Westrup B. (2017). Eight principles for patient-centred and family-centred care for newborns in the neonatal intensive care unit. Arch Dis Child Fetal Neonatal Ed.

[20] Davidson J.E., Aslakson R.A., Long A.C., Puntillo K.A., Kross E.K., Hart J. (2017). Guidelines for family-centered care in the neonatal, pediatric, and adult ICU. Crit Care Med.

[21] Larsen J.N., Hansson H., Beck S.A., Zoffmann V. (2024). Single-family rooms in neonatal intensive care: a qualitative analysis of fathers’, mothers’ and nurses’ experiences. J Neonatal Nurs.

[22] Wigert H., Dellenmark M.B., Bry K. (2013). Strengths and weaknesses of parent–staff communication in the NICU: a survey assessment. BMC Pediatr.

[23] Riskin A., Shlezinger S., Yonai L., Mor F., Partom L., Monacis-Winkler E. (2022). Improving communication with parents in the NICU during the COVID-19 pandemic, a study and review of the literature. Children.

[24] Brødsgaard A., Pedersen J.T., Larsen P., Weis J. (2019). Parents’ and nurses’ experiences of partnership in neonatal intensive care units: a qualitative review and meta-synthesis. J Clin Nurs.

[25] Lorié E.S., Willem-jan W.W., van Veenendaal N.R., van Kempen A.A., Labrie N.H. (2021). Parents’ needs and perceived gaps in communication with healthcare professionals in the neonatal (intensive) care unit: a qualitative interview study. Patient Educ Couns.

[26] Fazio S.B., Dany L., Dahan S., Tosello B. (2022). Communication, information, and the parent–caregiver relationship in neonatal intensive care units: a review of the literature. Arch Pediatr.

[27] Höglander J., Holmström I.K., Lövenmark A., Van Dulmen S., Eide H., Sundler A.J. (2023). Registered nurse–patient communication research: an integrative review for future directions in nursing research. J Adv Nurs.

[28] Herrick H.M., Wild K.T., Hill M. (2024). Video recording in neonatology: the need for objective measures and collaboration. Pediatr Res.

[29] Heath C., Hindmarsh J., Luff P. (2010).

[30] Golzar J., Noor S., Tajik O. (2022). Convenience sampling. Int J Educ Language Stud.

[31] Soni R., Tscherning C. (2021). Family-centred and developmental care on the neonatal unit. Paediatr Child Health.

[32] Klemming S., Lilliesköld S., Westrup B. (2021). Mother-newborn couplet care from theory to practice to ensure zero separation for all newborns. Acta Paediatr.

[33] Dore S., Fitzgerald F., Kelley K., Kuller J., Ludwig S., Peterman D. (2018). Applying developmentally supportive principles to diapering in the NICU: what we know. Neonatal Netw.

[34] Lyngstad L.T., Tandberg B.S., Storm H., Ekeberg B.L., Moen A. (2014). Does skin-to-skin contact reduce stress during diaper change in preterm infants?. Early Hum Dev.

[35] Bembich S., Fiani G., Strajn T., Sanesi C., Demarini S., Sanson G. (2017). Longitudinal responses to weighing and bathing procedures in preterm infants. J Perinat Neonatal Nurs.

[36] Jordan B., Henderson A. (1995). Interaction analysis: foundations and practice. J Learn Sci.

[37] Siltaloppi J., Vargo S.L. (2017). Triads: a review and analytical framework. Marketing Theory.

[38] Lincoln Y.S., Guba E.G. (1988).

[39] Malterud K., Siersma V.D., Guassora A.D. (2016). Sample size in qualitative interview studies: guided by information power. Qual Health Res.

[40] Karlsen M.-M.W., Sørensen K., Larsen B.H., Heyn L.G., Gerwing J. (2025). Video recording as a data collection method in vulnerable populations-methodological and ethical considerations. PEC Innov.

[41] Kerr D., Martin P., Furber L., Winterburn S., Milnes S., Nielsen A. (2022). Communication skills training for nurses: is it time for a standardised nursing model?. Patient Educ Couns.

[42] (2008). Lov om medisinsk og helsefaglig forskning (helseforskningsloven).

[43] Kocakabak C., Hoogen A.V.D., Rothfus M., Campbell-Yeo M., Abenstein A., Axelin A. (2026). Parents’ experiences and reported outcomes of family-centred care: a qualitative systematic review. Health Expect.

[44] Rholl E., Leuthner S.R., Malin K.J., Lagatta J., Olson K.R. (2025). “Ringleader who has no power”: a qualitative study of parent uncertainty during a NICU admission unrelated to prematurity, maternal health. Neonatol Perinatol.

[45] Fegran L., Helseth S. (2009). The parent–nurse relationship in the neonatal intensive care unit context–closeness and emotional involvement. Scand J Caring Sci.

[46] Lavallée A., Aita M., Côté J., Bell L., Luu T.M. (2020). A guided participation nursing intervention to theraupeutic positioning and care (GP_Posit) for mothers of preterm infants: protocol of a pilot randomized controlled trial. Pilot Feasibil Stud.

[47] Liu L.X., Mozafarinia M., Axelin A., Feeley N. (2019). Parents’ experiences of support in NICU single-family rooms. Neonatal Netw.

[48] Teixeira-Poit S.M., Fields B., Jenkins M., Jones S., Matthews C., Gharbi V. (2025). Nurse and parent perspectives of a neonatal intensive care unit redesign from open-bay to single-family rooms. J Perinatol.

[49] Yu C.C., Tan L., Le M.K., Tang B., Liaw S.Y., Tierney T. (2022). The development of empathy in the healthcare setting: a qualitative approach. BMC Med Educ.

[50] Petersson M.Å., Benzein E., Massoudi P., Wåhlin I., Persson C. (2023). Parents’ experiences of the significance of interpersonal interactions for becoming parents and a family during neonatal intensive care. J Pediatr Nurs.

[51] Tu T., Hu L., Yuan Y., Li H., Xie A., Guo H. (2025). A qualitative study of the emotional labour among neonatal nurses based on the affective events theory. Int J Qual Stud Health Well Being.

[52] Hagen L.W., Rød I., Kynø N., Tandberg B. (2025). Compassion fatigue among nurses in neonatal intensive care units. BMC Nurs.

[53] Elsharkawy N.B., Ramadan O.M.E., Hafiz A.H., Katooa N.E., Abunar A., Attallah D.M.A. (2026). Interactional compression and maternal participation in neonatal intensive care units: a qualitative study of nurse–mother communication barriers and co-produced solutions. Healthcare (Basel).

[54] Ottonello G., Rossi S., Dasso N., Da Rin Della Mora R., Calza S., Caracciolo G.M. (2025). The effects of the introduction of the single-family room in neonatal and paediatric intensive care on the outcomes of paediatric patients, families, staff, and organizations: a mixed method systematic review. BMC Health Serv Res.

[55] Gunduz S., Morgan C., Hoyle E., Turner M.A. (2026). Integrating family integrated care into neonatal practice: nursing experiences and education program development—a qualitative study. Front Pediatr.

[56] McNair C., McAllister M., Franck L.S., Stevens B., Taddio A. (2024). Parents’ experiences with infant pain management in the NICU. J Obstet Gynecol Neonatal Nurs.

